# Detection of *mec*A gene and methicillin-resistant *Staphylococcus aureus* (MRSA) isolated from milk and risk factors from farms in Probolinggo, Indonesia

**DOI:** 10.12688/f1000research.122225.1

**Published:** 2022-06-30

**Authors:** Aswin Rafif Khairullah, Saifur Rehman, Sri Agus Sudjarwo, Mustofa Helmi Effendi, Sancaka Chasyer Ramandinianto, Maria Aega Gololodo, Agus Widodo, Katty Hendriana Priscilia Riwu, Dyah Ayu Kurniawati

**Affiliations:** 1 Doctoral Program in Veterinary Science, Faculty of Veterinary Medicine, Universitas Airlangga, Surabaya, East Java, 60115, Indonesia; 2 Department of Veterinary Pharmacology, Faculty of Veterinary Medicine, Universitas Airlangga, Surabaya, East Java, 60115, Indonesia; 3 Department of Veterinary Public Health, Faculty of Veterinary Medicine, Universitas Airlangga, Surabaya, East Java, 60115, Indonesia; 4 Master Program in Veterinary Disease and Public Health, Faculty of Veterinary Medicine, Universitas Airlangga, Surabaya, East Java, 60115, Indonesia; 5 Department of Animal Infectious Diseases and Veterinary Public Health, Faculty of Medicine and Veterinary Medicine, Universitas Nusa Cendana, Kupang, Nusa Tenggara Timur, Indonesia

**Keywords:** Staphylococcus aureus, MRSA, Milk, Swab’s hand, Public health

## Abstract

**Background:** *Staphylococcus aureus* is commonly found in dairy cows and is a source of contamination in milk. *S. aureus* that are resistant to beta-lactam antibiotics are referred to as methicillin-resistant *Staphylococcus aureus* (MRSA). The spread of MRSA cannot be separated from sanitation management during milking; it can originate from milk collected from the udder or from the hands of farmers during the milking process. The purpose of this study was to examine the level of MRSA contamination in dairy cow's milk and farmer's hand swabs.

**Methods: **A total of 109 samples of dairy cow’s milk and 41 samples of farmers’ hand swabs were collected at a dairy farm in Probolinggo, East Java, Indonesia. Samples were cultured and purified using mannitol salt agar (MSA). The profile of *S. aureus* resistance was established by disk diffusion test using a disk of beta-lactam antibiotics, namely oxacillin and cefoxitin.

**Results: **The *S. aureus* isolates that were resistant to oxacillin and cefoxitin antibiotics were then tested for oxacillin resistance screening agar base (ORSAB) as a confirmation test for MRSA identity. *S. aureus* isolates suspected to be MRSA were then tested genotypically by polymerase chain reaction (PCR) method to detect the presence of the *mec*A gene. The results of the isolation and identification found 80 isolates (53.33%) of *S. aureus*. The results of the resistance test found that 42 isolates (15%) of *S. aureus* were resistant to oxacillin and 10 isolates (12.5%) were resistant to cefoxitin. The ORSAB test found as many as 20 isolates (47.62%) were positive for MRSA. In PCR testing to detect the presence of the *mec*A gene, three isolates (30%) were positive for the *mec*A gene.

**Conclusions: **This study shows that several *S. aureus* isolates were MRSA and had the gene encoding *mec*A in dairy farms.

## Introduction

*Staphylococcus aureus* is a pathogenic bacteria that can cause public health problems, because these bacteria often contaminate products of animal origin, including milk or commonly known as milk-borne disease (MBD). ^1^ *S. aureus* is an opportunistic bacterial pathogen that can be found in animals and humans. This bacterium can cause various diseases ranging from mild to systemic skin infections such as pneumonia, arthritis, and meningitis. ^2^ In previous studies, *S. aureus* was mostly transmitted to humans through contaminated milk. ^3^ *S. aureus* is commonly found on the skin and mucosa of livestock, especially dairy cows with subclinical or clinical mastitis, which is a source of contamination in milk. ^4^ These bacteria can quickly evolve into antibiotic-resistant strains due to inappropriate antibiotic administration. ^5^ *S. aureus* that is resistant to beta-lactam antibiotics is referred to as methicillin-resistant *S. aureus* (MRSA). ^6^

In previous studies, it was reported that the presence of MRSA can cause new health problems for humans and animals. ^7^ The high rate of MRSA contamination in dairy farms due to excessive administration of antibiotics in the treatment of dairy cows and the spread of these bacteria cannot be separated from sanitation management during milking. Contamination can happen from milk that is collected from the udder as well as from the hands of farmers during the milking process. ^8^ The Probolinggo Regency, specifically in Krucil District, is one of the largest milk-producing centers in Indonesia. ^9^ Antibiotics have been widely used as treatment in cases of infection in dairy cattle in Probolinggo, especially in cases of mastitis, so contamination by MRSA in dairy farms in Probolinggo ^10^ is possible.

*S. aureus* evolved into strain MRSA because it received the insertion of a large DNA element between 20-100 kb called staphylococcal cassette chromosome *mec* (SCC *mec*), that underlies the change in normal penicillin-binding protein (PBP), namely PBP2 to PBP2a. ^11^ PBP2a is expressed by the gene encoding *mec*A contained in SCC *mec* which has a very low affinity for beta-lactams, so that even t cultured on media containing high concentrations of beta-lactams, MRSA survives. ^12^ Molecular detection of the *mec*A gene using polymerase chain reaction (PCR) is often carried out to confirm the presence of MRSA isolates, but cannot be done in all laboratories because of the ability and cost constraints. ^13^ Constraints in the use of PCR can be replaced by examining MRSA using the disk diffusion method with the antibiotics oxacillin and cefoxitin, which is then continued with an examination using oxacillin resistance screening agar base (ORSAB). ^14^

The purpose of this study was to examine the level of MRSA contamination in dairy cow’s milk and farmer’s hand swab in Probolinggo, Indonesia, as well as to compare phenotypic detection methods using screening with oxacillin and cefoxitine diffusion disks, ORSAB, and confirming genotypes using PCR to detect *mec*A-coding genes. The sensitivity and specificity of the test show the effectiveness and ease of application of the MRSA detection method.

## Methods

### Sampling

A total of 109 samples of dairy cow’s milk and 41 samples of farmer’s hand swabs were collected at a dairy farm in the Probolinggo region, East Java, Indonesia from July to September 2021. Dairy cow’s milk samples were taken from each cow in the third press as much as 30 mL which was then stored in a 60 mL sample bottle; the farmer’s hand swab samples were taken from each farmer after the milking process using a sterile cotton swab which was then stored on Amies medium.

### Bacteria isolation and identification

As much as 1 mL of each milk sample was put into a 20 mL test tube filled with 9 mL of mannitol salt broth (MSB) medium while for hand swab samples, the Amies medium was vortexed until it became liquid and then 1 mL was added into a 20 mL test tube which has been filled with 9 mL of MSB media. The test tube containing MSB which had been mixed with the sample was incubated in an incubator (Isuzu Model 2-2195, Jica) at 37°C for 24 hours. The samples were cultured and purified using mannitol salt agar (MSA) (Oxoid CM0085) and then incubated at 37°C for 24 hours.

Microscopic examination of bacteria was done through Gram staining to visualise Gram-positive bacteria in the form of cocci and clusters. ^15^ The biochemical examination was carried out using a catalase test and a coagulase test. The catalase test was carried out by dripping 3% hydrogen peroxide (H _2_O _2_) on bacterial colonies that had been placed on the surface of the glass. ^16^ The coagulase test was carried out by dripping 200 μl of rabbit plasma into a coagulase test tube containing bacterial colonies, which was then incubated at 37°C for 24 hours. ^17^

### Oxacillin and cefoxitin disk diffusion methods

The test was carried out following the Clinical and Laboratory Standards Institute (CLSI) 2020 guidelines: *S. aureus* was tested for susceptibility to the antibiotics oxacillin 30 μg and cefoxitin 30 μg (Oxoid) on Muller Hinton Agar (MHA) plates (Oxoid, CM0337). The identified isolates were purified on mannitol salt agar (HiMedia Pvt. Ltd., M118), incubated at 37°C for 24 hours as a 0.5 Mc Farland suspension, and then taken using a sterile cotton swab of size S (AKD 10903610549). They were then wiped evenly on the surface of the MHA medium (Oxoid, CM0337). diskThe oxacillin 30 μg and cefoxitin 30 μg antibiotic disks were placed side by side with a distance of 5 cm on MHA that had been inoculated with isolates, and then incubated at 37°C for 24 hours to measure the inhibition zone.

### Oxacillin resistance screen agar test

*S. aureus* isolates resistant to oxacillin 30 μg and cefoxitin 30 μg (Oxoid) were confirmed by ORSAB (HiMedia M1415) using *S. aureus* isolates from the MH media; plus Oxacillin Resistance Selective Supplement (Supplement, HiMedia Pvt. Ltd., FD191). ^18^

### Detection of the *mecA* gene

All *S. aureus* isolates that were resistant to cefoxitin 30 μg and positive on ORSAB examination were then subjected to a PCR test to detect the presence of the *mec*A gene. ^19^ The DNA extraction process was carried out according to the QIAamp DNA Mini Kit protocol (51304 & 51306), where previously the isolates were purified on MSA (HiMedia Pvt. Ltd, M118) and inoculated on MHA (Oxoid, CM0337). The primer used was *mec*A F: 5′-AAA ATC GAT GGT AAA GGT TGG C-3′ and *mec*A R: 5′-AGT TCT GCA GTA CCG GAT TTG C-3′. ^20^ The PCR master mix used GoTaq Green Master Mix (Promega, 9PIM712) which is a ready-to-use solution mixture containing Taq DNA polymerase, dNTPs, MgCl _2_, and a reaction buffer. DNA was amplified using a Thermal Cycler T100 machine (Bio-Rad, 186-1096) for 40 cycles in 25 μL of the reaction mixture with the following steps: denaturation at 94°C for 30 seconds, annealing at 55°C for 30 seconds, and extension at 72°C for 1 min with a final extension at 72°C for 5 min. A total of 10 μL of PCR product were analyzed by 2% agarose gel electrophoresis, and the gel was visualized under ultraviolet light. ^21^ A positive test indicated a PCR product in the 533-base pair (bp) band.

## Result

**Figure 1.**
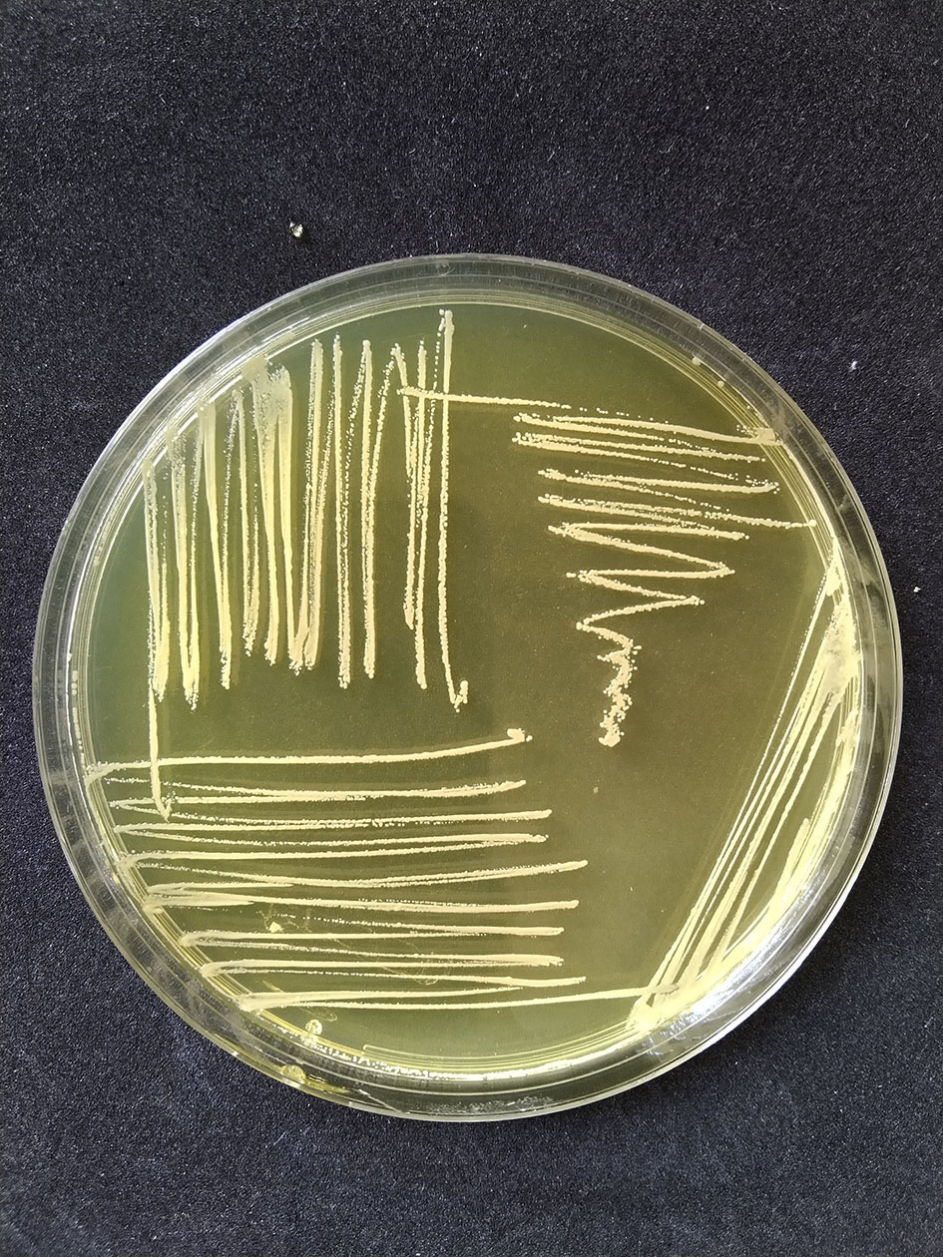
The results of positive yellow mannitol fermentation and *Staphylococcus aureus* colonies appear mucoid white on mannitol salt agar (MSA) medium (HiMedia Pvt. Ltd, M118).

The results of the isolation and identification tests yielded 80 (53.33%) *S. aureus* isolates from 150 samples taken at a dairy farm in Probolinggo, East Java, Indonesia. The 80 isolates that were positive for *S. aureus* consisted of 54 isolates from dairy cow’s milk samples and 26 isolates from farmers’ hand swab samples as shown in Table 1. *S. aureus* had phenotypic colony characteristics on MSA medium, namely a change in color in the medium from red to yellow indicating mannitol fermentation, while the colonies had various pigments including white, orange, and yellow as shown in Figure 1. The Gram staining test showed the Gram-positive colonies in the form of cocci and clusters, which were then confirmed by the catalase test and coagulase test. ^16^

**Table 1.** Isolation of *Staphylococcus aureus* by type of sample.

Sample type	Sample code	Sample size	Positive *S. aureus* (%)
Milk	AS	109	54 (49.54%)
Swab hand	AT	41	26 (63.41%)
**Total**		**150**	**80 (53.33%)**

Note: % = Percentage of positive *Staphylococcus aureus*.

**Figure 2.**
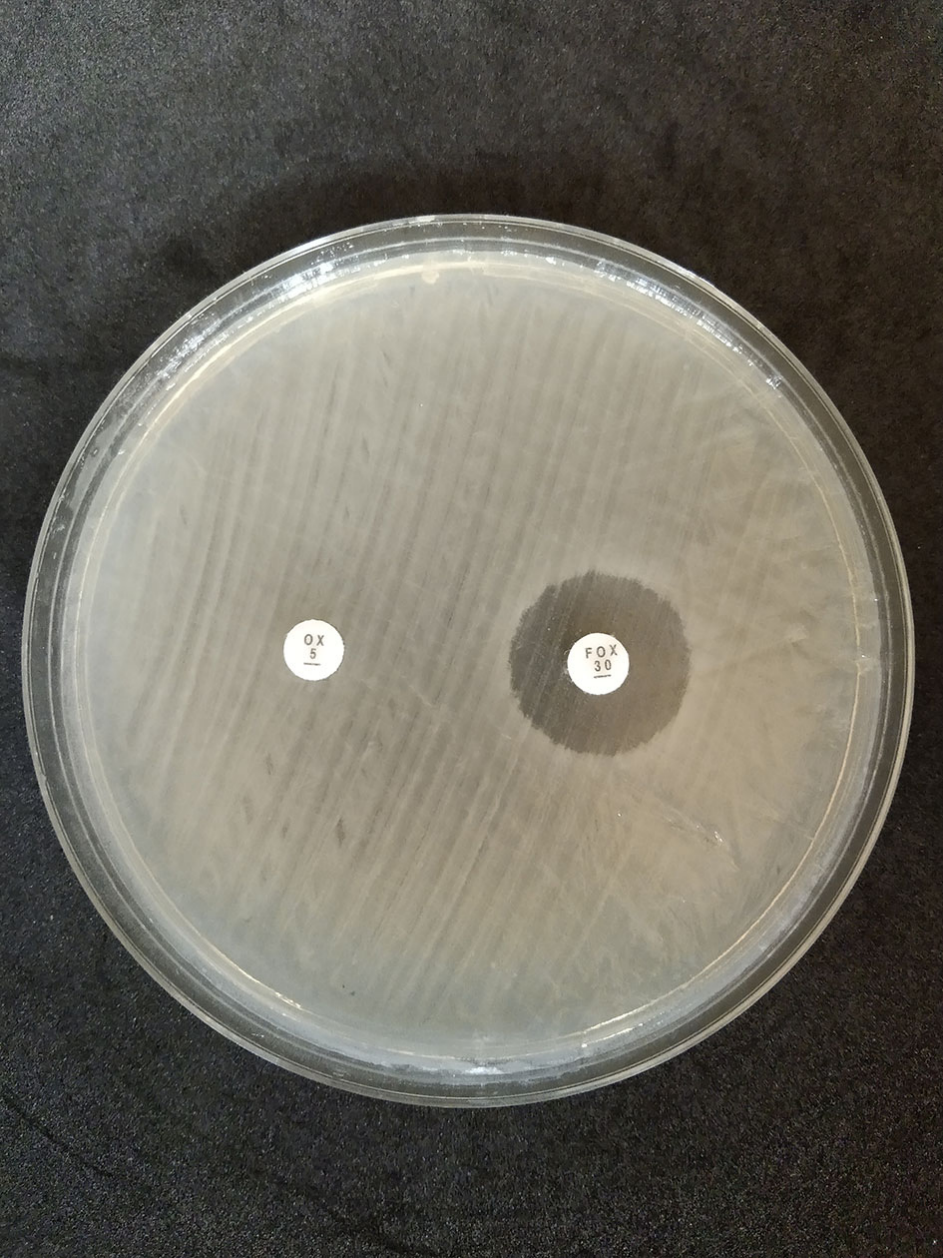
Oxacillin (OX) and cefoxitin (FOX) resistant to disk diffusion test in Mueller Hinton Agar (MHA) (Oxoid, CM0337); OX = oxacillin, FOX = cefoxitin.

The disk diffusion method on MHA medium showed that 42 isolates exhibited resistance to oxacillin preparations, with a percentage of 52.5% (28 isolates came from dairy cow’s milk samples and 14 isolates came from farmers’ hand swab sample); on the other hand, 10 isolates showed resistance to cefoxitin, with a percentage of 12.5% (five isolates came from dairy cow’s milk samples and five isolates came from farmers’ hand swab samples) as shown in Table 2 and Figure 2.

**Table 2.** Oxacillin and cefoxitin disk diffusion test of *Staphylococcus aureus.*

Sample type	*Staphylococcus aureus* isolate (n=80)			
	OX disk diffusion		FOX disk diffusion	
	Resistant (%)	Sensitive (%)	Resistant (%)	Sensitive (%)
Milk	28 (35%)	26 (32.5%)	5 (6.25%)	49 (61.25)
Swab hand	14 (17.5%)	12 (15%)	5 (6.25%)	21 (26.25%)
**Total**	**42 (52.5%)**	**38 (47.5%)**	**10 (12.5%)**	**70 (87.5%)**

Note: % (Percentage), Total percentage of positive or resistant resistant values in *Staphylococcus aureus* isolates; OX = oxacillin 30 μg, FOX = cefoxitin 30 μg (Oxoid).

Based on the results of the disk diffusion test, no *S. aureus* isolate was to only be resistant to cefoxitin: all *S. aureus* isolates that were detected to be resistant to cefoxitin were also identified as resistant to oxacillin as shown in Table 3.

**Table 3.** Positive MRSA confirmed by oxacillin and cefoxitin disk diffusion, ORSAB and *mec*A gene detection.

Sample type	Sample code	Resistance to disk diffusion test		ORSAB Test	*mec*A detection using PCR	Number positive of MRSA isolates by *mec*A detection (%)
		OX	FOX			
Milk	AT 21	+	+	+		2 (20%)
	AT 28	+	+	+		
	AT 29	+	+	+		
	AT 33	+	+	+		
	AT 41	+	+	+		
Swab hand	AS 67	+	+	+		1 (10%)
	AS 77	+	+	+		
	AS 80	+	+	+		
	AS 102	+	+	+		
	AS 109	+	+	+		
Total						3 (30%)

Note = OX: oxacillin 30 μg, FOX: cefoxitin 30 μg (Oxoid); % (percentage): Total percentage of *Staphylococcus aureus* isolates positive for MRSA identification by PCR at the sampling location; +: Resistant.

Confirmation of the phenotype test that for resistance to oxacillin and cefoxitin was followed by ORSAB test, with a blue culture coloration indicating positive results while a white coloration indicated negative results. The ORSAB test showed that of the 42 isolates of *S. aureus* that were resistant to oxacillin, 20 isolates (47.62%) were confirmed MRSA by the disk diffusion method, as shown in Table 4.

**Table 4.** Total number confirmed MRSA by oxacillin resistance screening agar base (ORSAB).

Sample type	Sample code	Number of isolates tested ORSAB (n=42)	Positive ORSAB test
Milk	AS	28 (66.67%)	15 (35.71%)
Hand swab	AT	14 (33.33%)	5 (11.9%)
Total		42 (100%)	20 (47.62%)

Note: % (Percentage).

**Figure 3.**
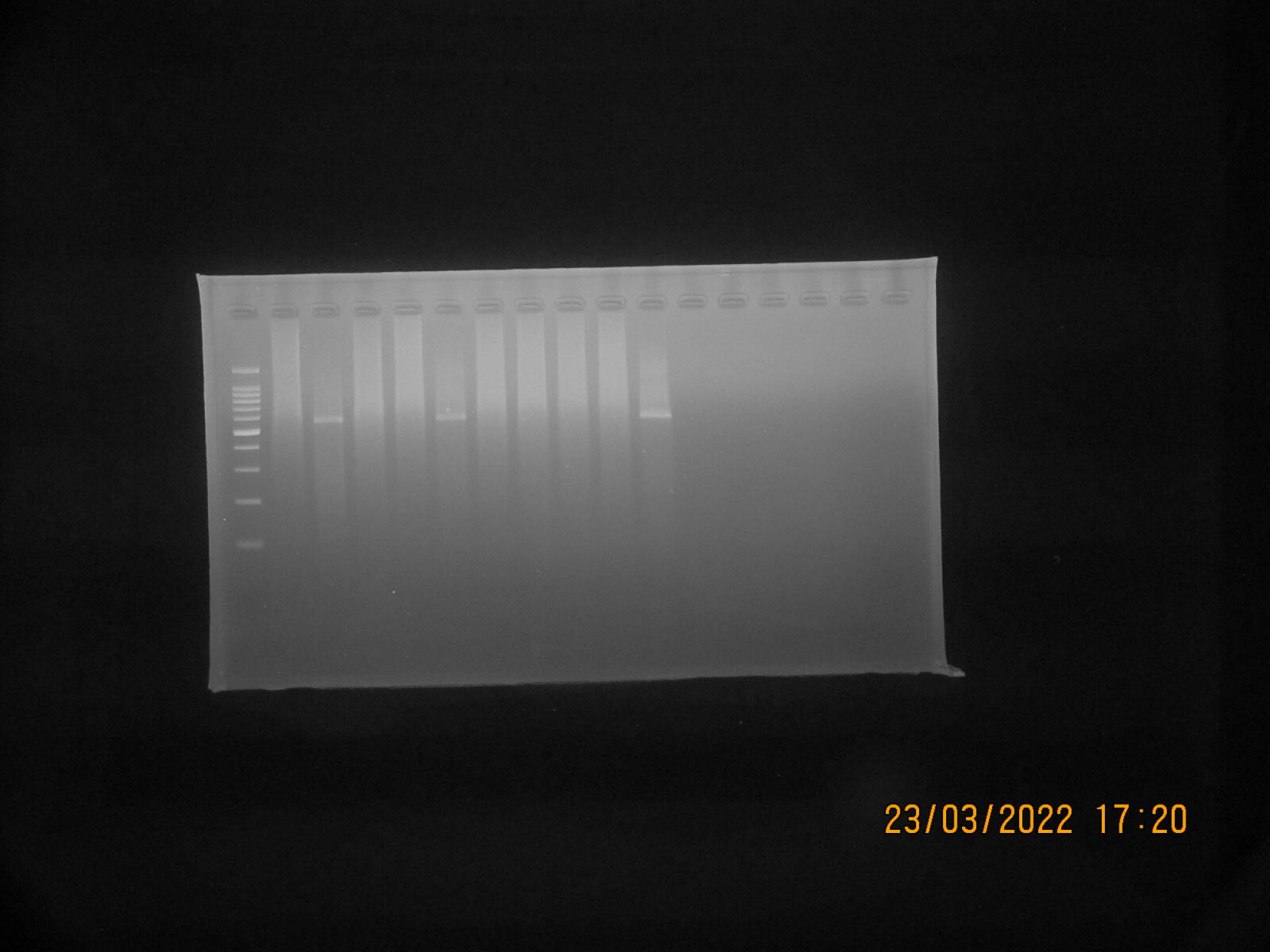
Detection *mec*A PCR results with positive bands at 533 bp; Marker line: 100-bp molecular-weight markers; Line K-: *Staphylococcus aureus* ATCC 25923 (Negative Control); Line AT28, AT41, and AS109: Positive result for *mec*A gene; Line AT21, AT29, AT33, AS67, AS77, AS80, and AS102: Negative result for *mec*A gene.

*S. aureus* isolates suspected to be MRSA (Phenotypically resistant to cefoxitin and positive for ORSAB) were then tested genotypically using PCR to detect the presence of the gene encoding *mec*A. A total of 10 isolates suspected to be MRSA were tested, from which three isolates (30% of the total isolates tested by PCR) were detected positive for the *mec*A gene, as shown in Figure 3. The results of the PCR test showed that isolates suspected to be MRSA were found to have the *mec*A gene, which is resistant to the antibiotics cefoxitin and oxacillin, as shown in Table 3.

## Discussion

MBD is quite a common public health problem, because it not only has an impact on human health, also has an impact on the health of dairy cows, especially in the milk production and quality sector. ^22^ Several previous studies have reported that the incidence of contaminated milk by *S. aureus* resistant to antibiotics is found in both developed and developing countries. ^23^ Improper and unhygienic handling of milk, especially during the milking process, plays an important role in the occurrence of milk contamination. ^24^ Unhygienic farmer hands when milking can also potentially transmit pathogenic bacteria in milk, including *S. aureus*. ^25^

*S. aureus* is a pathogenic bacterium that can cause various infectious diseases ranging from skin infections to systemic infections that can lead to death. ^26^ In this study, of 150 milk samples, 80 samples (53.33%) were found to have *S. aureus* contamination; this percentage is higher than the research conducted by Wang *et al.* ^27^ which isolated 195 milk samples, of which 90 samples (46.15%) were contaminated with *S. aureus*, and from another study conducted by Jahan *et al.* ^28^ who isolated 47 milk samples, of which 12 (25.53%) were contaminated with *S. aureus.* This study employed a purposive sampling design that was carried out to detect the presence of *S. aureus* strains in dairy farms that have low milking hygiene, which can increase bacterial contamination in cow’s milk. ^29^ In line with this, the research conducted by Khiabanian *et al.* ^30^ showed that the difference in the number of isolates found could be influenced by differences in study design such as population and geographic distribution of the sample, infection control practices, and the type of antibiotic used, as seen in Figure 3 *mec*A PCR results with a positive band at 533 bp. Marker line: 100-bp molecular-weight markers; Line K-: *Staphylococcus aureus* ATCC 25923 (Negative Control); Line AT28, AT41, and AS109: positive result for *mec*A gene detection; Line AT21, AT29, AT33, AS67, AS77, AS80, and AS102: negative result for *mec*A gene.

The problem of the incidence of *S. aureus* infection continues to grow with the emergence of MRSA, which is resistant to all beta-lactam antibiotics, including monobactams and cephalosporins, which are a group of antibiotics often used to treat *Staphylococcus* infections. ^31^ MRSA infection causes treatment problems and facilitates its spread, so prompt and early diagnosis is needed to identify MRSA accurately. ^32^ In this study, 42 samples (52.5%) of *S. aureus* were found to be resistant to oxacillin disks, and 10 samples (12.5%) to cefoxitin disks. Miragaia ^33^ stated that the phenotypic detection of MRSA using disk diffusion still has not shown accurate results, and *mec*A genotyping using PCR is still the main recommendation even though it cannot be done routinely. However, even so, identification of MRSA with disk diffusion is still widely used because it can be done quickly and at a lower cost. ^34^ Diffusion disks using Oxacillin and Cefoxitin have the same sensitivity level of 100%, and specificities of 74.07% for oxacillin and 92.59% for cefoxitin. ^35^ However, several previous studies reported that the use of the cefoxitin disk diffusion method had a better sensitivity level than that of oxacillin in detecting MRSA, because the oxacillin disk diffusion method still has a high false positive rate. ^36^ Vyas *et al.* ^35^ stated that false positives could be influenced by beta-lactamase hyperproduction, resulting in the phenotypic expression of oxacillin resistance but without a genotypic resistance mechanism.

In this study, all isolates detected were resistant to the cefoxitin and oxacillin disks. All isolates detected to be resistant to oxacillin and cefoxitin were confirmed by ORSAB assay, in line with a report by Pourmand *et al.* ^37^ which stated that the ORSAB test has a specificity of 100%. In this study, 20 of the 42 isolates (47.62%) were found to be positive for MRSA. The sensitivity level confirmed the resistance strain being tested while the specificity was to the minimum inhibitory concentration (MIC). ^38^ Cefoxitin-resistant and ORSAB-positive *S. aureus* isolates were tested genotypically using PCR to detect the presence of the gene encoding *mec*A; these isolates also had positive results in all phenotypic methods (resistance to cefoxitin and oxacillin in the disk diffusion method and positive results in the ORSAB test). These results are similar to those from research conducted by Ramandinianto *et al.* ^39^ The antibiotic cefoxitin is a good inducer for the expression of the *mec*A gene because it can increase the expression of PBP2a, which is encoded by the *mec*A gene. ^40^ This also agrees with Reichmann and Pinho ^41^ and Anand *et al.* ^42^

From this study, it can be concluded that the occurrence of MRSA contamination in milk can be caused by various factorsincluding the unhygienic hands of farmers when milking. ^43^ MRSA contamination poses a serious public health risk, which increases the potential for the spread of difficult-to-treat staphylococci. ^44^ Therefore, microbiology laboratory examinations are very important to isolate and identify MRSA isolates quickly, accurately, and cost-effectively from food samples of animal origin. ^45^ Genotypic detection using PCR to detect the presence of the gene encoding *mec*A is a molecularly accurate MRSA test; however, in laboratories that cannot perform molecular testing, the cefoxitin disk diffusion method can be used to A. ^46^ This is based on the ability of the cefoxitin disk diffusion test in detecting the expression of the *mec*A gene which can be a more effective and efficient MRSA screening method. ^47^

## Conclusions

This study shows that several *S. aureus* isolates are Methicillin-Resistant *S. aureus* (MRSA) and have the gene encoding *mec*A in dairy farms. The spread of *S. aureus* that is MRSA can be a threat to public health. Thus, prevention and control measures are needed to suppress the spread of *S. aureus* infection on a dairy farm in Probolinggo, East Java, Indonesia.

## Data availability

### Underlying data

Figshare: Detection of mecA gene and methicillin-resistant Staphylococcus aureus (MRSA) isolated from milk and risk factors from the farmer in Probolinggo, Indonesia, https://doi.org/10.6084/m9.figshare.19784005.

This project contains the following underlying data:

- CMT data and code sample (Argopuro).xlsx
- Results of Isolation and Identification (Argopuro).xlsx
- Bacterial resistance test results (Argopuro).xlsx
- MRSA confirmatory test results (Argopuro).xlsx

### Extended data

Figshare: Detection of mecA gene and methicillin-resistant Staphylococcus aureus (MRSA) isolated from milk and risk factors from the farmer in Probolinggo, Indonesia, https://doi.org/10.6084/m9.figshare.19784005.

This project contains the following extended data:

- Table and Figure.docx

Data are available under the terms of the Creative Commons Attribution 4.0 International license (CC-BY 4.0).
