# Supplementary material for: Detection of mecA gene and methicillin-resistant Staphylococcus aureus (MRSA) isolated from milk and risk factors from farms in Probolinggo, Indonesia
Source: F1000Res. 2022 Sep 20;11:722. Originally published 2022 Jun 30. [Version 3] doi: 10.12688/f1000research.122225.3 (PMC9607882; doi:10.12688/f1000research.122225.3)
Supplement: Peer review history

# Peer review: Reviewer response for version 3

**DOI:** 10.5256/f1000research.138608.r150894

**Published:** 2022-09-20

**Authors:** F M Yasir Hasib

**Affiliations:** 1 Department of Infectious Diseases and Public Health, Jockey Club College of Veterinary Medicine and Life Sciences, City University of Hong Kong, Hong Kong, China

Thank you for the revised version of the manuscript. I think the authors did a great job in addressing all the comments related to the initial review.

Is the work clearly and accurately presented and does it cite the current literature?

Yes

If applicable, is the statistical analysis and its interpretation appropriate?

Yes

Are all the source data underlying the results available to ensure full reproducibility?

Yes

Is the study design appropriate and is the work technically sound?

Partly

Are the conclusions drawn adequately supported by the results?

Yes

Are sufficient details of methods and analysis provided to allow replication by others?

Yes

Reviewer Expertise:

Antibiotic resistance, Public health, Bioinformatics

I confirm that I have read this submission and believe that I have an appropriate level of expertise to confirm that it is of an acceptable scientific standard.

# Peer review: Reviewer response for version 2

**DOI:** 10.5256/f1000research.138347.r150170

**Published:** 2022-09-13

**Authors:** Ikechukwu Benjamin Moses

**Affiliations:** 1 Department of Applied Microbiology, Faculty of Science, Ebonyi State University, Abakaliki, Nigeria; 2 Division of Infectious Diseases, Escola Paulista de Medicina, Federal University of Sao Paulo, Sao Paulo, Brazil

My concerns have been addressed by the authors.

Is the work clearly and accurately presented and does it cite the current literature?

Yes

If applicable, is the statistical analysis and its interpretation appropriate?

Not applicable

Are all the source data underlying the results available to ensure full reproducibility?

Yes

Is the study design appropriate and is the work technically sound?

Partly

Are the conclusions drawn adequately supported by the results?

Yes

Are sufficient details of methods and analysis provided to allow replication by others?

Yes

Reviewer Expertise:

Antimicrobial resistance surveillance in veterinary and human medicine, and the development of alternative therapeutic approaches.

I confirm that I have read this submission and believe that I have an appropriate level of expertise to confirm that it is of an acceptable scientific standard.

# Peer review: Reviewer response for version 1

**DOI:** 10.5256/f1000research.134188.r149189

**Published:** 2022-09-05

**Authors:** Ikechukwu Benjamin Moses

**Affiliations:** 1 Department of Applied Microbiology, Faculty of Science, Ebonyi State University, Abakaliki, Nigeria; 2 Division of Infectious Diseases, Escola Paulista de Medicina, Federal University of Sao Paulo, Sao Paulo, Brazil

**General comments:**

 The article entitled “ *Detection of mecA gene and methicillin-resistant Staphylococcus aureus (MRSA) isolated from milk and risk factors from farms in Probolinggo, Indonesia*” was well-written. The study design and objectives of the manuscript were good and address a critical public health issue, especially with regards to foodborne illnesses and the spread of clinically-relevant bacterial pathogens (MRSA) in livestock and their by-products. However, the authors need to make a few grammatical and technical corrections which I have already suggested in my attached comments. The research findings are in very good tandem with the research aim and objectives. If the authors are able to make the few corrections I suggested, it can be indexed.

**Abstract:**

 The abstract is good and well-structured but needs a few corrections:

- *Sentence 1*: “ *S. aureus *that are resistant to beta-lactam antibiotics are referred to as methicillin-resistant *Staphylococcus aureus *(MRSA).”
**Comment 1**: Authors should rephrase/restructure the above sentence in the abstract section because beta-lactam antibiotics include penicillin, ampicillin, etc. So, if for example, an isolate is resistant to penicillin or even ampicillin, it can’t be referred to as methicillin-resistant. The disc diffusion method using cefoxitin disc (30 µg) is more reliable in identifying methicillin-resistant *S*. *aureus* (MRSA). So, I suggest that authors should re-write this sentence as “ *S. aureus *that are resistant to beta-lactam antibiotics **(especially cefoxitin)** are referred to as methicillin-resistant *Staphylococcus aureus *(MRSA).
- *Sentence 2* **:** “The purpose of this study was to examine the level of MRSA contamination in dairy cow's milk and farmer's hand swabs.”
**Comment 2**: Authors should delete the word “swabs” in the sentence because what is being actually assessed are the hands of the farmers. The swab is just a tool used to collect the sample.
**Comment 3:** The keyword " **Swab's hand"** should be changed to **"hand swabs" **in the list of keywords.

**Introduction:**

 The introduction was generally very good. I will suggest that the authors make a change in the last paragraph of this section:

- **Last paragraph of introduction: **The purpose of this study was to examine the level of MRSA contamination in dairy cow’s milk and farmer’s hand **swab** in Probolinggo, Indonesia, as well as to compare phenotypic detection methods using screening with oxacillin and cefoxitine diffusion disks, ORSAB, and confirming genotypes using PCR to detect mecA-coding genes.
**Comment:** I think the authors should remove the word “swab” as what is being actually assessed are the **farmers’ hands**, just like I mentioned in my earlier suggestion in the abstract section.

**Methods:**

 The methodology was well-detailed except for some important technical corrections which I have suggested:

- **Oxacillin and cefoxitin disk diffusion methods**
  The test was carried out following the Clinical and Laboratory Standards Institute (CLSI) 2020 guidelines: S. aureus was tested for susceptibility to the antibiotics **oxacillin 30 μg** and cefoxitin 30 μg (Oxoid) on Muller Hinton Agar (MHA) plates (Oxoid, CM0337). The identified isolates were purified on mannitol salt agar (HiMedia Pvt. Ltd., M118) **, **incubated at 37°C for 24 hours **as a 0.5 McFarland suspension, and then taken using a sterile cotton swab of size S (AKD 10903610549). They were then wiped **evenly on the surface of the MHA medium (Oxoid, CM0337) **. Disk. **The oxacillin **30 μg **and cefoxitin 30 μg antibiotic disks were placed side by side with a distance of **5 cm **on MHA that had been inoculated with isolates, and then incubated at 37°C for 24 hours to measure the inhibition zone.
**Comment 1**: Authors should correct the concentration of oxacillin antibiotic disc to **1** **μg** because oxacillin disc concentration from Oxoid, UK is **1** **μg **while that of cefoxitin is correct at the **30** **μg **indicated. I think this might have been an oversight during the writing of the manuscript.
**Comment 2**: Authors should take note of the **bolded sections** in the sentence and make corrections as I indicated below for the sentence to be more comprehensive and understandable. Also, 5cm is the same as 50mm, so it is preferable to indicate that the distance between the oxacillin and cefoxitin antibiotics was 50 mm instead of 5 cm since distance units in the CLSI charts are in mm. As I mentioned earlier, the sentence in the last section should be written as: “The identified isolates were purified on mannitol salt agar (HiMedia Pvt. Ltd., M118) **and **incubated at 37°C for 24 hours **. Using a sterile cotton swab** (AKD 10903610549), **standardized isolates (0.5 McFarland standard) were evenly streaked** on the surface of the MHA medium (Oxoid, CM0337). The oxacillin **(1 μg)** and cefoxitin **(30 μg)** antibiotic disks were placed side by side with a distance of 50 mm on MHA that had been inoculated with isolates, and then incubated at 37°C for 24 hours to measure the inhibition zone.”
**Comment 3**: The concentration of all the oxacillin discs in the manuscript should be changed to **1 μg**.

**Results:**

 The results are very clear and understandable. Data were properly interpreted and comprehensive. However, I suggested some important changes and corrections:

- **Comment 1:** The colour of *S*. *aureus* on mannitol salt agar (MSA) is golden-yellow. I will suggest authors use this all through the manuscript.
- *Sentence*: “Based on the results of the disk diffusion test, no S. aureus isolate was to only be resistant to cefoxitin: all S. aureus isolates that were detected to be resistant to cefoxitin were also identified as resistant to oxacillin as shown in Table 3.”
**Comment 2:** I suggest that authors should rephrase this sentence to be more understandable.
**Comment 3**: I suggest that the authors delete the column “ **mecA detection using PCR**” **in Table 3 **as it is empty and serves no function since the last column is already indicating the total isolates that harboured the mecA gene.

**Discussion:**

 The discussion is good but needs some critical changes in some confusing sentences which I have suggested below:

- *Sentence*:  ..contamination; this percentage is higher than the research conducted by Wang et al. ^27^ which isolated 195 milk samples, of which 90 samples (46.15%) were contaminated with S. aureus, and from another study conducted by Jahan et al. ^28^ who isolated 47 milk samples, of which 12 (25.53%) were contaminated with S. aureus.
**Comment 1**: There is a mix-up in the sentence above. The sentence is stating that milk samples were isolated while what was actually isolated was the *S. aureus* from the milk samples. I will suggest that authors should re-write this section as “:  ..contamination; this percentage is higher than the research conducted by Wang *et al*. ^27^ **who isolated 90 (46.15 %) *S*. *aureus* from 195 milk samples, and from another study conducted by Jahan *et al*. ^28^ who isolated 12 (25.53%) *S*. *aureus* from 47 milk samples**”.
- *Sentence*: …mecA PCR results with a positive band at 533 bp. Marker line: 100-bp molecular-weight markers; Line K-: Staphylococcus aureus ATCC 25923 (Negative Control); Line AT28, AT41, and AS109: positive result for mecAgene detection; Line AT21, AT29, AT33, AS67, AS77, AS80, and AS102: negative result for mecAgene.
**Comment 2:** I think the authors should delete this sentence since Figure 3 already has text under the gel picture depicting what was positive for mecA, including the targeted fragment size and what was negative.

Is the work clearly and accurately presented and does it cite the current literature?

Yes

If applicable, is the statistical analysis and its interpretation appropriate?

Not applicable

Are all the source data underlying the results available to ensure full reproducibility?

Yes

Is the study design appropriate and is the work technically sound?

Partly

Are the conclusions drawn adequately supported by the results?

Yes

Are sufficient details of methods and analysis provided to allow replication by others?

Yes

Reviewer Expertise:

Antimicrobial resistance surveillance in veterinary and human medicine, and the development of alternative therapeutic approaches.

I confirm that I have read this submission and believe that I have an appropriate level of expertise to confirm that it is of an acceptable scientific standard, however I have significant reservations, as outlined above.

# Response

**Published:** 2022-09-06

**Authors:** Aswin Rafif Khairullah

**Affiliations:** Universitas Airlangga, Indonesia

Respected reviewer Moses, thanks a lot for your able input. We have followed all your kind suggestions accordingly. Kindly find our response:

 Thanks for your valuable comments and suggestions on the manuscript entitled:

 “ **Detection of *mec*A gene and methicillin-resistant *Staphylococcus aureus* (MRSA) isolated from milk and risk factors from farms in Probolinggo, Indonesia”**

 We welcome feedback. We have made modifications to the study on the following points:

**Abstract section:**

 Authors should rephrase/restructure the above sentence in the abstract section because beta-lactam antibiotics include penicillin, ampicillin, etc. So, if for example, an isolate is resistant to penicillin or even ampicillin, it can’t be referred to as methicillin-resistant. The disc diffusion method using cefoxitin disc (30 μg) is more reliable in identifying methicillin-resistant S. aureus (MRSA). So, I suggest that authors should re-write this sentence as “S. aureus that are resistant to beta-lactam antibiotics (especially cefoxitin) are referred to as methicillin-resistant Staphylococcus aureus (MRSA).

**Response:** We have revised and rephrased/restructured this sentence: " *S. aureus *that are resistant to beta-lactam antibiotics are referred to as methicillin-resistant *Staphylococcus aureus *(MRSA).”

 Authors should delete the word “swabs” in the sentence because what is being actually assessed are the hands of the farmers. The swab is just a tool used to collect the sample.

**Response:** We have deleted the word “swabs” in the sentence.

**Introduction section**

 I think authors should remove the word “swab” as what is being actually assessed are the **farmers’ hands **just like I mentioned in my earlier suggestion in the abstract section.

**Response:** We have removed the word swabs accordingly as you suggested to us.

**Methodology section**

 Authors should correct the concentration of oxacillin antibiotic disc to **1 μg **because oxacillin disc concentration from Oxoid, UK is **1 μg **while that of cefoxitin is correct at the 30 **μg **indicated. I think this might have been an oversight during the writing of the manuscript.

**Response: **According to your suggestion we have corrected the concentration of oxacillin antibiotic disc to **1 μg.**

 Authors should take note of the **bolded sections **in the sentence and make corrections as I indicated below for the sentence to be more comprehensive and understandable. Also, 5cm is the same as 50mm. So, it is preferable to indicate that the distance between the oxacillin and cefoxitin antibiotics were 50 mm instead of 5 cm since distance units in the CLSI charts are in mm. Like I mentioned earlier, the sentence in the last section should be written as: “The identified isolates were purified on mannitol salt agar (HiMedia Pvt. Ltd., M118) **and **incubated at 37°C for 24 hours **. Using a sterile cotton swab **(AKD 10903610549), **standardized isolates (0.5 McFarland standard) were evenly streaked **on the surface of the MHA medium (Oxoid, CM0337). The oxacillin **(1 μg) **and cefoxitin **(30 μg) **antibiotic disks were placed side by side with a distance of 50 mm on MHA that had been inoculated with isolates, and then incubated at 37°C for 24 hours to measure the inhibition zone.”

**Response: **We have corrected the whole section accordingly as you suggested. The concentration of all the oxacillin discs in the manuscript should be changed to **1μg**.

**Results section**

 The colour of *S*. *aureus *on mannitol salt agar (MSA) is golden-yellow. I will suggest authors use this all through the manuscript.

**Response: **We have corrected this sentence throughout the manuscript.

 I suggest that authors should rephrase this sentence to be more understandable.

**Response: **We have rephrased the whole sentence of table three so that it is understandable.

 I suggest that authors delete the column of “ **mecA detection using PCR**” **in Table 3 **as it is empty and serves no function since the last column is already indicating the total isolates that harboured mecA gene

**Response: **We have followed your suggestions and made changes in Table 3.

**Discussion section**

 There is a mix up in the sentence above. The sentence is stating that milk samples were isolated while what was actually isolated were the *S. aureus *from the milk samples. I will suggest that authors should re-write this section as “: ..contamination; this percentage is higher than the research conducted by Wang *et al*.27 **who isolated 90 (46.15 %) *S*. *aureus *from 195 milk samples, and from another study conducted by Jahan *et al*.** **28** **who isolated 12 (25.53%) *S*. *aureus *from 47 milk samples**”.

**Response: **We have corrected your recommended sentence accordingly in the whole section of the manuscript.

 I think Authors should delete this sentence since Figure 3 already has a writing under the gel picture depicting what was positive for mecA, including the targeted fragment size and what as negative.

**Response: **We have deleted this sentence.

# Peer review: Reviewer response for version 1

**DOI:** 10.5256/f1000research.134188.r143155

**Published:** 2022-07-26

**Authors:** F M Yasir Hasib

**Affiliations:** 1 Department of Infectious Diseases and Public Health, Jockey Club College of Veterinary Medicine and Life Sciences, City University of Hong Kong, Hong Kong, China

**Keywords:**

1. No need for 'Swab's hand' as a keyword.

**Introduction:**

1. " *Staphylococcus aureus *is a pathogenic bacteria...", " *S. aureus* is an opportunistic bacterial pathogen..." - The two lines are almost similar and seem redundant. However, using pathogenic bacteria on the first line is unnecessary.
2. "In previous studies, *S. aureus* mainly was transmitted to humans through contaminated milk..." - In this line, you should add at least three references.
3. "These bacteria can quickly evolve into antibiotic-resistant strains due to inappropriate antibiotic administration" - Please delete the line or re-write it. It's challenging to make such a bold statement regarding drug administration.
4. " *S. aureus* that is resistant to beta-lactam antibiotics is referred to as methicillin-resistant *S. aureus* (MRSA)" - Why this line again? Please check the coherence of the lines.
5. "In previous studies, it was reported that the presence of MRSA can cause new health problems..." - Please re-write the introduction section to improve the flow of the introduction - sorry.

**Methods:**

1. How was the sample size calculated?
2. Please describe the sampling strategy.
3. What was the condition of the cows? i.e., lactation, age, antibiotic administration, housing type, mastitis condition, etc.
4. How do you select the dairy animals from the herd?
5. Why were hand swabs taken after milking? Before milking seems more accurate. Before milk collection, what process did you follow for the aseptic milk collection?
6. I don't find any importance in microscopic examination here.
7. What was the positive isolate used as a standard?
8. For MRSA, you can do only Cefoxitin disk diffusion for the confirmation. Oxacillin MIC is recommended according to CLSI (13th edition, CLSI, M02, Page 30 ^1^).

**Results:**

1. "The Gram staining test showed the Gram-positive colonies in the form of cocci and clusters, which were then confirmed by the catalase test and coagulase test" - Change the line and add both biochemical test results (+/-).
2. You did not include the correlation between the isolates recovered from milk or hand swab.
3. "...milk can be caused by various factors including the unhygienic hands of farmers when milking..." - You did not take the swab before milking, how do you argue about it?

**Discussion and Conclusions:**

1. I think the authors did a great job describing the findings.

Is the work clearly and accurately presented and does it cite the current literature?

Yes

If applicable, is the statistical analysis and its interpretation appropriate?

Yes

Are all the source data underlying the results available to ensure full reproducibility?

Yes

Is the study design appropriate and is the work technically sound?

Partly

Are the conclusions drawn adequately supported by the results?

Yes

Are sufficient details of methods and analysis provided to allow replication by others?

Yes

Reviewer Expertise:

Antibiotic resistance, Public health, Bioinformatics

I confirm that I have read this submission and believe that I have an appropriate level of expertise to confirm that it is of an acceptable scientific standard, however I have significant reservations, as outlined above.

## References

[1] : M02 Performance Standards for Antimicrobial Disk Susceptibility Tests, 13th Edition.2018;30 Reference source

# Response

**Published:** 2022-09-13

**Authors:** Aswin Rafif Khairullah

**Affiliations:** Universitas Airlangga, Indonesia

Thanks for your valuable comments and suggestions on the manuscript entitled:

 “ **Detection of *mec*A gene and methicillin-resistant *Staphylococcus aureus* (MRSA) isolated from milk and risk factors from farms in Probolinggo, Indonesia”**

**Abstract section:**

 No need for 'Swab's hand' as a keyword.

**Response:** We have removed the "swab hand's" description in the keywords.

**Introduction section**

 " *Staphylococcus aureus* is a pathogenic bacteria...", " *S. aureus* is an opportunistic bacterial pathogen..." - The two lines are almost similar and seem redundant. However, using pathogenic bacteria on the first line is unnecessary.

**Response:** We have removed the description of *S. aureus* in the second sentence so that there is no repetition of the word.

 " *Staphylococcus aureus* is a pathogenic bacteria...", " *S. aureus* is an opportunistic bacterial pathogen..." - The two lines are almost similar and seem redundant. However, using pathogenic bacteria on the first line is unnecessary.

**Response:** We have removed the description of *S. aureus* in the second sentence so that there is no repetition of the word.

 "In previous studies, *S. aureus* mainly was transmitted to humans through contaminated milk..." - In this line, you should add at least three references.

**Response:** We have added two more references to the sentence so that there are three references in the sentence.

 "These bacteria can quickly evolve into antibiotic-resistant strains due to inappropriate antibiotic administration" - Please delete the line or re-write it. It's challenging to make such a bold statement regarding drug administration.

**Response:** We have deleted the sentence.

 " *S. aureus* that is resistant to beta-lactam antibiotics is referred to as methicillin-resistant *S. aureus* (MRSA)" - Why this line again? Please check the coherence of the lines.

**Response:** We have corrected the sentence to make it clearer.

 "In previous studies, it was reported that the presence of MRSA can cause new health problems..." - Please re-write the introduction section to improve the flow of the introduction

**Response:** We have corrected the sentence to make it clearer.

**Methodology section**

 How was the sample size calculated?

**Response:** We have added the sample calculation formula to the methodology

 Please describe the sampling strategy.

**Response:** Based on these calculations, a sample was obtained with the selection of dairy cooperatives purposively based on the amount of milk production in an area and the willingness of dairy cooperatives to participate in the study.

 What was the condition of the cows? i.e., lactation, age, antibiotic administration, housing type, mastitis condition, etc.

**Response:** Milk samples were taken from the udders of female cows who were in the lactation period.

 How do you select the dairy animals from the herd?

**Response:** The condition of most dairy farms in Indonesia is not in a herd, but a few dairy cows are kept in cages.

 Why were hand swabs taken after milking? Before milking seems more accurate. Before milk collection, what process did you follow for the aseptic milk collection?

**Response:** Because after the milking process there is cross-contamination of MRSA transmission from cows to humans and humans to cows. We have sterilized the sample bottles that we will use to take samples and milk samples taken directly from the cow's nipples.

 I don't find any importance in microscopic examination here.

**Response:** We have added a description of the microscopic examination in Table 1 along with a picture of the results of Gram staining observed through a microscope.

 What was the positive isolate used as a standard?

**Response:** Isolates were tested positive for *Staphylococcus aureus* when they had yellow colonies on Mannitol Salt Agar (MSA) media accompanied by positive results on Gram-positive staining and biochemical tests (catalase test and coagulase test).

 For MRSA, you can do only Cefoxitin disk diffusion for the confirmation. Oxacillin MIC is recommended according to CLSI (13th edition, CLSI, M02, Page 301).

**Response:** Because cefoxitin has a higher sensitivity and specificity than oxacillin in detecting MRSA.

**Results section**

 "The Gram staining test showed the Gram-positive colonies in the form of cocci and clusters, which were then confirmed by the catalase test and coagulase test" - Change the line and add both biochemical test results (+/-).

**Response:** We have added the description of the Gram stain examination, catalase test, and coagulase test in Table 1 along with pictures.

 You did not include the correlation between the isolates recovered from milk or hand swab.

**Response:** Because in this study we focused on observing risk factors for MRSA transmission originating from dairy cows.

 "...milk can be caused by various factors including the unhygienic hands of farmers when milking..." - You did not take the swab before milking, how do you argue about it?

**Response:** Because we had difficulty in sampling on the farms, especially when sampling farmers' hand swabs due to the large number of milk samples we took. Besides that, it was very difficult to get samples of farmers' hand swabs before milking on several farms at the same time.
